# (DEAD)-box RNA helicase 3 modulates NF-κB signal pathway by controlling the phosphorylation of PP2A-C subunit

**DOI:** 10.18632/oncotarget.16593

**Published:** 2017-03-27

**Authors:** Xin Wang, Rui Wang, Miao Luo, Chen Li, Hua-Xia Wang, Chang-Chao Huan, Yu-Rong Qu, Ying Liao, Xiang Mao

**Affiliations:** ^1^ College of Veterinary Medicine, Nanjing Agricultural University, Nanjing, 210095, China; ^2^ Shanghai Veterinary Research Institute, Chinese Academy of Agricultural Sciences, Shanghai, 200241, China

**Keywords:** (DEAD)-box RNA helicase 3, PP2A, NF-κB

## Abstract

Asp-Glu-Ala-Asp (DEAD)-box RNA helicase 3 (DDX3), an ATP-dependent RNA helicase, is associated with RNA splicing, mRNA export, transcription, translation, and RNA decay. Recent studies revealed that DDX3 participates in innate immune response during virus infection by interacting with TBK1 and regulating the production of IFN-β. In our studies, we demonstrated that DDX3 regulated NF-κB signal pathway. We found that DDX3 knockdown reduced the phosphorylation of p65 and IKK-β and ultimately attenuated the production of inflammatory cytokines induced by poly(I:C) or TNF-α stimulation. The regulatory effect of DDX3 on NF-κB signal pathway was not affected by the loss of its ATPase or helicase activity. We further identified PP2A C subunit (PP2A-C) as an interaction partner of DDX3 by co-immunoprecipitation and mass spectrum analysis. We confirmed that DDX3 formed the complex with PP2A-C/IKK-β and regulated the interaction between IKK-β and PP2A-C. Furthermore, we demonstrated that DDX3 modulated the activity of PP2A by controlling the phosphorylation of PP2A-C, which might enable PP2A-C to regulate NF-κB signal pathway by dephosphorylating IKK-β. All these findings suggested DDX3 plays multiple roles in modulating innate immune system.

## INTRODUCTION

The helicases are nucleic acid-dependent ATPases, which utilize the energy provided by nucleoside triphosphate hydrolysis to unwind and remodel DNA or RNA [1–3]. The helicases can be classified as DNA or RNA helicases based on their substrates; some act on both DNA and RNA molecules [3]. DNA helicases participate in many DNA metabolic processes like DNA repair, replication, recombination and telomere maintenance [4–6], whereas RNA helicases are involved in almost all aspects of RNA metabolism including RNA maturation and splicing, mRNA export, transcription, translation, and RNA decay [7–9].

The DEAD-box and the related DEAH, DExH and DExD RNA helicases are members of superfamily II; they share seven to eight conserved motifs that are involved in ATP binding, ATP hydrolysis, nucleic acid binding, and RNA unwinding activity [10–13]. The DEAD-box protein family is named by one of the motifs (Asp-Glu-Ala-Asp, or DEAD) [11]. The DEAD-box helicases are ubiquitously expressed in all eukaryotes and most prokaryotes and they participate in various RNA metabolic processes like other RNA helicases [14–16].

DDX3 is a member of the DEAD-box RNA helicase family [17]. DDX3 has two homologs, DDX3X and DDX3Y. The gene of DDX3X located in Xp11.3–p11.23 while the DDX3Y is located in the non-recombining region of the Y chromosome (Yq11) [17, 18]. Although they share high amino acid similarity (91.7%), DDX3X cannot rescue the loss-of-function of DDX3Y, suggesting the functional divergence between DDX3X and DDX3Y [19, 20]. DDX3Y is detected only in the male germ line, whereas DDX3X protein is expressed in all tissues analyzed [21]. DDX3(X) gene encodes a polypeptide of 661 or 662 residues [22, 23]. DDX3 mostly accumulates in the cytoplasm in resting state. Meanwhile, it can also act as a nucleo-cytoplasmic shuttling protein. For example, DDX3 associates with CRM1 and assist HIV Rev-RRE export. DDX3 can also interact with another nuclear export factors TAP that helps mRNPs export [24, 25]. Expect its role in RNA metabolism, DDX3 can also participate in cell cycle progression, apoptosis and cancer [23, 26–31].

Accumulating studies indicate that DDX3 contributes to innate immune response, either by acting as the viral nucleic acid sensor or by facilitating downstream signaling events. For example, DDX3 affects the production of IFN-β through its interaction with TBK1 and IKKε [9, 32]. DDX3 enhances the auto-phosphorylation of IKKε by directly interacting with IKKε. DDX3 phosphorylation at serine 102 (S102) by IKKε is required for recruitment of IRF3 to DDX3, after which IRF3 can be phosphorylated by IKKε and subsequently activates IFN-β signal pathway [33]. DDX3 C-terminal region can directly bind to IPS-1 CARD-like domain and enhances the IPS-1 mediated IFN-β promoter activation [34].

As a transcription factor, NF-κB controls the expression of a wide variety of genes involved in inflammatory factors and cell survival [35]. In mammal cells, the NF-κB family has five transcription factors: RelA (p65), RelB, c-Rel, p50/p105 (NF-κB1) and p52/p100(NF-κB2). All of them share a highly conserved DNA-binding/dimerization domain in their N-terminus [36]. In most un-stimulated cells, NF-κB exists as a protein complex by combining with each other to form homodimers or heterodimers. The p50/p65 heterodimer is the major NF-kB dimer, which is involved in regulation of the innate immune response and cell survival [37]. P65 contains the transcriptional activation domain and can regulate anti-apoptotic gene expression [38–40]. The p50 is usually considered to act as a transcriptional repressor when binding κB sites as the homodimer [41, 42]. NF-κB mainly distributes in the cytoplasm by interacting with the inhibitory IκB proteins (IκBα, IκBβ, IκBε and Bcl-3) [43]. The canonical NF-κB can be activated by pro-inflammatory cytokines, bacterial or viral antigens [44–46]. When activated, the IKK-β subunit is first activated and leads to the phosphorylation and degradation of IκB proteins. After IκB protein is degraded, the complexes such as p50/p65, p50/c-Rel are released and translocate to the nucleus to regulate the expression of downstream genes [47]. NF-κB plays an important role in regulating cellular differentiation, survival and proliferation, inflammation and innate immunity.

In eukaryotic cells, various cellular functions are controlled by the subtle shift between protein phosphorylation and dephosphorylation, which are strictly regulated by kinases and phosphatases. At least 518 human kinases have been confirmed, including 385 protein-serine/threonine kinases, 90 protein-tyrosine kinases and 43 tyrosine-kinase like proteins [48–50]. However, the number of protein phosphatases (30 serine/threonine phosphatases and 107 tyrosine phosphatases) is much smaller than that of kinases [51, 52]. The protein phosphatase 2A (PP2A), a major serine/threonine phosphatase which accounts for almost 1% of the total cellular protein and 80% of total serine/threonine phosphatase [53], has been reported to regulate many cellular events such as proliferation, survival and apoptosis [54]. PP2A consists of three subunits: scaffold A(PP2A-A), regulatory B (PP2A-B), and catalytic C subunit (PP2A-C). The scaffold A and catalytic C subunits interact to form the core enzyme. The regulatory B subunit has at least 18 isoforms These regulatory B subunits play the key roles in controlling PP2A substrate specificity, cellular localization, and enzymatic activity [56]. As a protein phosphatase, PP2A is involved in regulating many signaling pathways like Akt, p53, c-Myc and β-catenin [57–61]. Okadaic acid, the specific PP2A inhibitor, activates NF-κB [62]. The IκB kinase complex (IKK), critical for IκB phosphorylation and NF-κB activation, is sensitive to PP2A and can be inactivated by PP2A [63]. PP2A catalytic subunit interacts with IKK-β, thereby negatively regulates NF-κB signal pathway [64]. PP2A can also interact with p65 in melanocytes in the absence of stimulation, suggesting p65 can be dephosphorylated by PP2A-C in the cells [65]. All these reports show that PP2A negatively regulates NF-κB signal pathway.

In our studies, we found that the knockdown of DDX3 reduced the phosphorylation of p65 and IKK-β, which ultimately reduced the production of inflammatory cytokines. We identified PP2A C subunit (PP2A-C) as an interaction partner of DDX3 by co-immunoprecipitation and mass spectrum analysis. We confirmed that DDX3 formed the complex with PP2A-C/IKK-β and regulated the interaction between IKK-β and PP2A-C. Our experiments also revealed that DDX3 modulated the activity of PP2A by controlling the phosphorylation of PP2A-C, which might enable PP2A-C to regulate NF-κB signal pathway by dephosphorylating IKK-β. All these findings suggested DDX3 plays multiple roles in modulating innate immune system, except for its roles in IFN-β signal pathway.

## RESULTS

### The knockdown of DDX3 reduces the production of inflammatory cytokines

**Figure 1:**
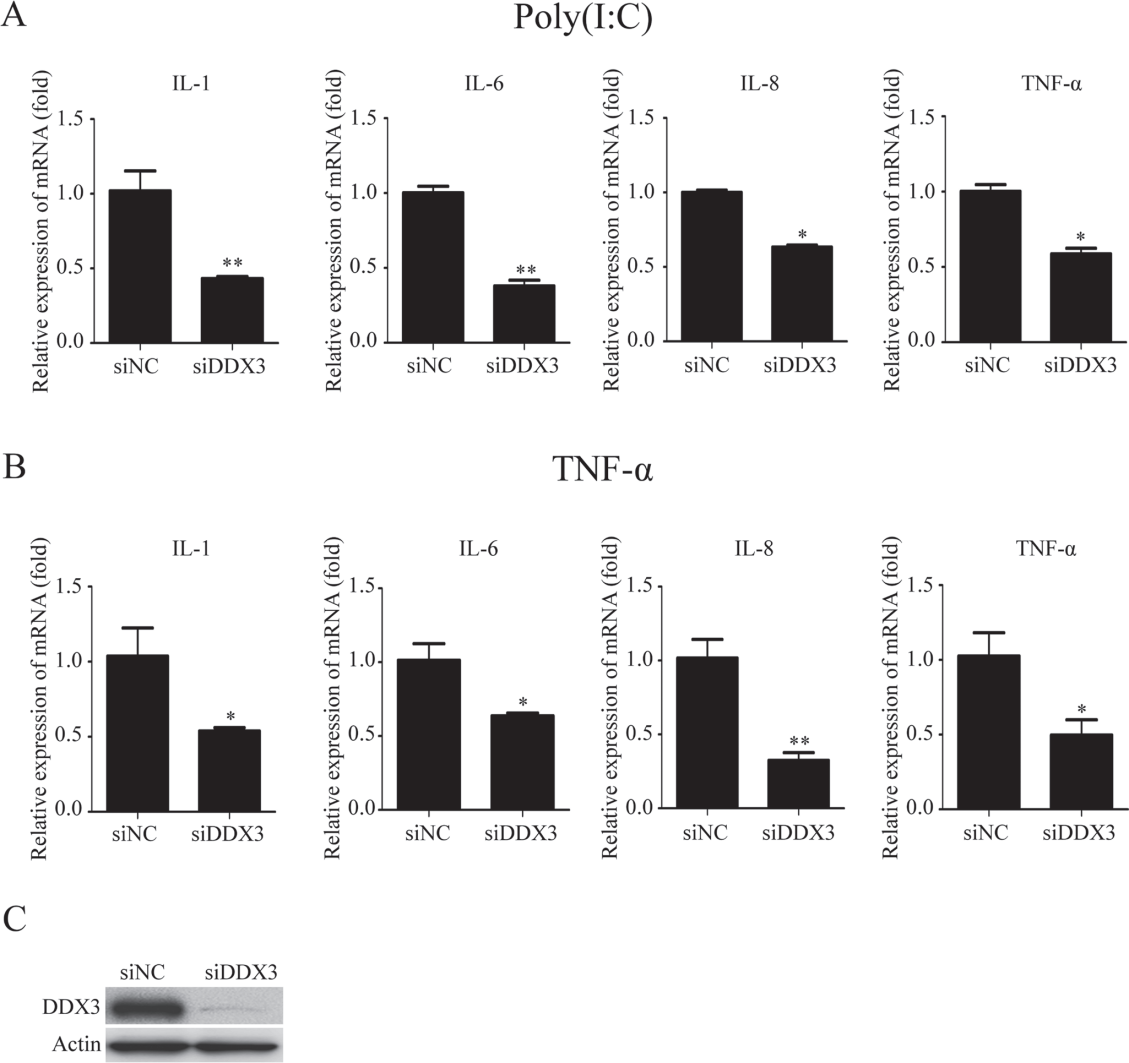
The knockdown of DDX3 reduces the production of proinflammatory cytokines induced by poly(I:C) and TNF-α HeLa cells were transfected with siDDX3 (small interfering RNA specifically targeting DDX3) for 48 hours before transfected with poly(I:C) (5 μg/ml) for 6 hours (**A**) or stimulated with TNF-α (25 ng/ml) for 6 hours (**B**). Total RNA was extracted from the cells and the relative amounts of IL-1b, IL-6, IL-8, TNF-α mRNA was assessed by qRT-PCR. The scrambled siRNA (siNC) were used as the control. The differences between means were considered significant at *p < 0.05, very significant **p < 0.01. The DDX3 RNA knockdown effect was detected by western blot analysis (siDDX3) (**C**).

The previous studies have revealed that DDX3 affects the production of IFN-β due to its interaction with TBK1 and IKKε [9, 32]. The cytokine production is one of the important indicators of the activation of the innate immune responses [66]. In order to study whether DDX3 affected the production of the proinflammatory cytokines, we detected the production of several inflammatory cytokines using qRT-PCR after the expression of DDX3 in HeLa cells (human cervical cancer cell line) was silenced with small interfering RNA (siRNA) specific targeting DDX3 (siDDX3). Poly(I:C) is a synthetic analog of double-stranded RNA (dsRNA), a mimic of the pathogen-associated molecular pattern. Many proteins like TLR3, RIG-I/MDA5 and PKR recognize poly(I:C), which would activate the innate immune response, including NF-κB signal pathway [67, 68]. Meanwhile, tumor necrosis factor (TNF) is also one of the most potent physiological activators of the nuclear transcription factor NF-κB [69]. Therefore, in our studies, we used poly(I:C) and TNF-α to induce the activation of NF-κB signal pathway. The qRT-PCR results showed that the mRNA levels of IL-1β, IL-6, IL-8 and TNF-α were decreased about 57%, 62%, 37% and 41% in the cells stimulated with poly(I:C) (Figure 1A). Meanwhile, the mRNA levels of IL-1β, IL-6, IL-8, TNF-α were decreased about 46%, 38%, 74%, and 64% in the cells stimulated with TNF-α (Figure 1B). Western blot analysis exhibited an efficient knockdown of endogenous DDX3 expression compared with cells transfected with scrambled siRNA (siNC) (Figure 1C). These results suggested that DDX3 affected the production of proinflammatory cytokines.

### DDX3 regulates the NF-κB activation

**Figure 2:**
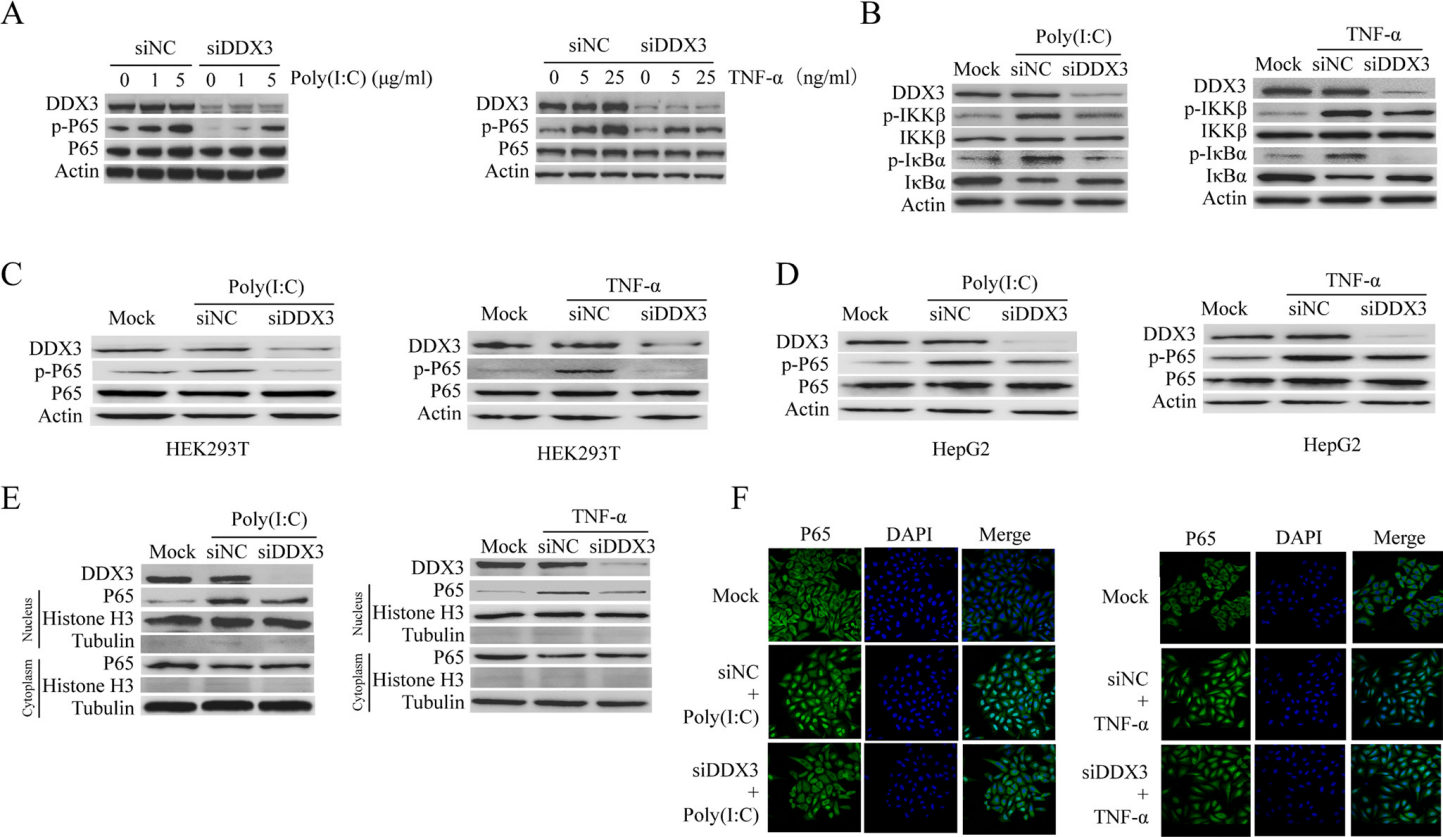
DDX3 regulates NF-κB signal pathway HeLa cells were first transfected with siDDX3 for 48 hours to silence DDX3 expression before the phosphorylation of p65 was tested by western blot after the cells were either transfected with Poly(I:C)(1 μg/ml or 5 μg/ml) for 6 hours or stimulated with TNF-α (5 ng/ml or 25 ng/ml) for 6 hours (**A**). The same experiment was also performed in HEK-293T (**C**) and HepG2 cells (**D**). The phosphorylation of IκBα and IKK-β, as well as the degradation of IκBα, were also determined after the cells were treated by 5 μg/ml poly(I:C) or 25 ng/ml TNF-α (**B**). The level of p65 in the cytoplasm and nucleus were detected after HeLa cells were stimulated with poly(I:C) or TNF-α (**E**). For IFA detection, HeLa cells were first seeded on glass cover slips for 24 hours before transfected with siDDX3 for 48 hours to silence DDX3 expression. After stimulated with poly(I:C) (5 μg/ml) or TNF-α (25 ng/ml) for 1 hour, the cells were fixed and immunostained for p65 using a rabbit anti-p65 antibody and Alexa Fluor^®^ 488 goat anti-rabbit IgG (H+L) secondary antibody. Nuclei were counterstained with DAPI. The images were captured by confocal microscopy (**F**). The scrambled siRNAs (siNC) were used as the control.

It is well-known that cytokine production can be regulated by the NF-κB signal pathway [66]. Meanwhile, both poly(I:C) and TNF-a could activate NF-κB signal pathway. Therefore, we speculated that DDX3 might regulate inflammatory cytokine production through NF-κB. We first tested whether DDX3 knockdown affected the phosphorylation of p65, the critical process for NF-κB pathway activation. Western blot analysis showed that the phosphorylation of p65 in HeLa cells stimulated by poly(I:C) or TNF-α was decreased after DDX3 expression was knocked down (Figure 2A). The same results were also observed in HEK-293T (human embryonic kidney cell line) (Figure 2C) and HepG2 cells (human liver cancer cell line) (Figure 2D).

P65 translocates into the nucleus to activate the transcription of multiple target genes after IκBα phosphorylation and degradation [70, 71]. Our data revealed that the phosphorylation and degradation of IκBα were attenuated in DDX3 knockdown cells (Figure 2B). The catalytic activity of IKK-β contributes essentially to IκBα phosphorylation and NF-kB activation [66, 72, 73]. Our results also confirmed that DDX3 knockdown reduced the phosphorylation of the upstream signaling molecule IKK-β (Figure 2B).

To confirm the above results, we stimulated the DDX3 knockdown cells with poly(I:C) or TNF-α and analyzed the protein levels of p65 in the nuclear and cytoplasmic separately, which showed that the protein level of p65 in the nucleus was decreased. Meanwhile, there was no cross-contamination between nuclear and cytoplasmic extracts as determined by nuclear and cytoplasmic markers histone H3 and tubulin (Figure 2E). The same results were also obtained by IFA which showed that the nuclear translocation of p65 was reduced after DDX3 knockdown (Figure 2F). All these results suggested that DDX3 regulated the NF-κB signal pathway.

### The ATPase and helicase activities of DDX3 do not influence NF-κB signal

**Figure 3:**
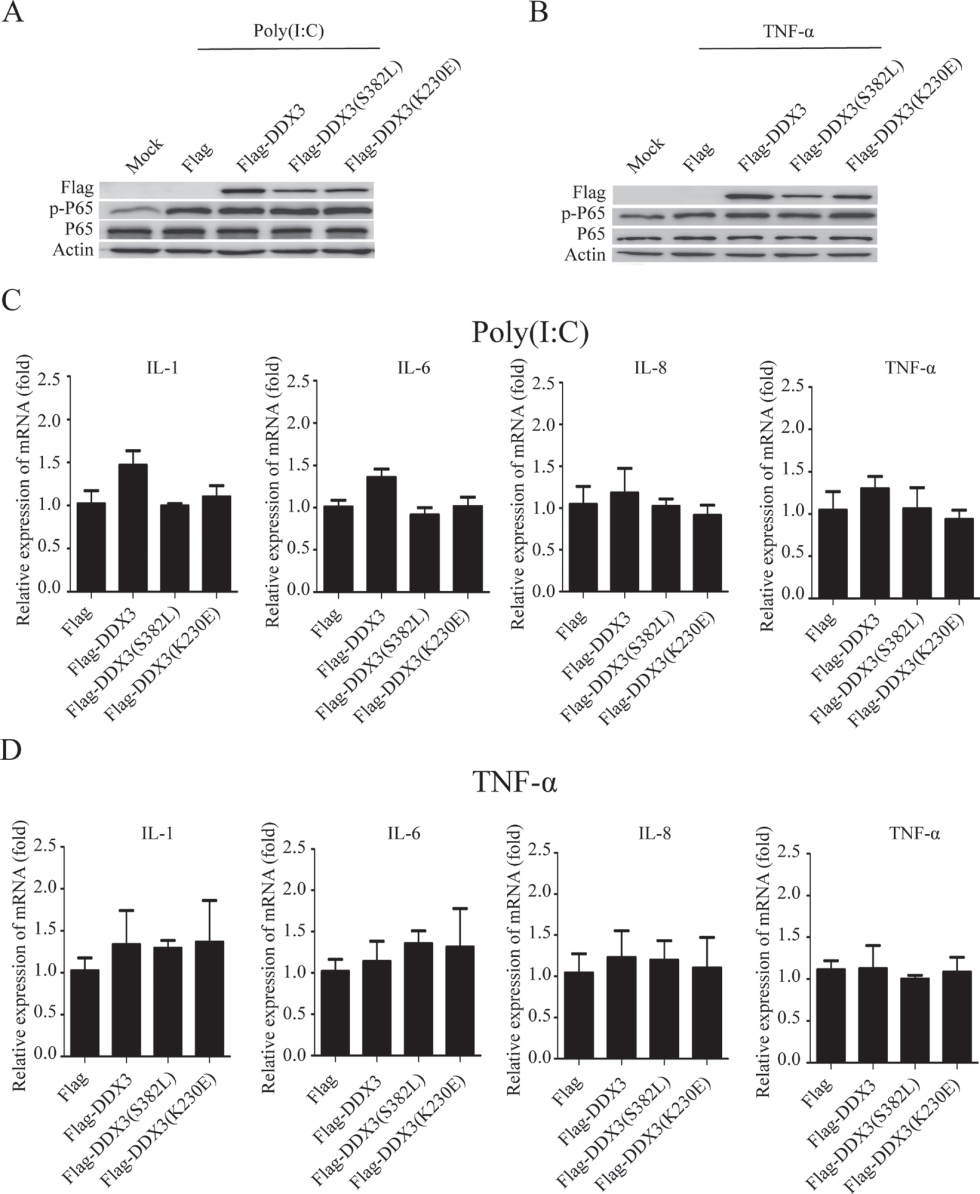
The ATPase and helicase activities of DDX3 do not influence NF-κB signal pathway HeLa cells were first transfected with the plasmids: Flag, Flag-DDX3, Flag-DDX3(S382L), Flag-DDX3(K230E) for 48 hours then were stimulated with poly(I:C) or TNF-α for another 6 hours. The phosphorylation of p65 was detected by western blot (**A, B**) and the relative expression of IL-1, IL-6, IL-8, TNF-α mRNA was assessed by qRT-PCR(**C, D**). Data are representative of at least two independent experiments.

As a member of the DEAD-box helicase family, DDX3 exerts its functions mostly depend on its ATPase and helicase activities. We, therefore, constructed wild-type DDX3 (Flag-DDX3) as well as helicase-dead DDX3 mutant either lacking the ATPase activity (K230E) or RNA unwinding activity (S382L) [8, 24, 32, 74]. HeLa cells were transfected with these plasmids, and the phosphorylation of p65 was detected after the cells were stimulated with poly(I:C) or TNF-α. The over-expression of neither wild-type nor the mutant DDX3 proteins affected the phosphorylation of p65 (Figure 3A, 3B). On the other hand, the production of cytokines was also changed little as determined from the results of the qRT-PCR (Figure 3C, 3D).

### DDX3 interacts and co-localizes with PP2A-C

**Figure 4:**
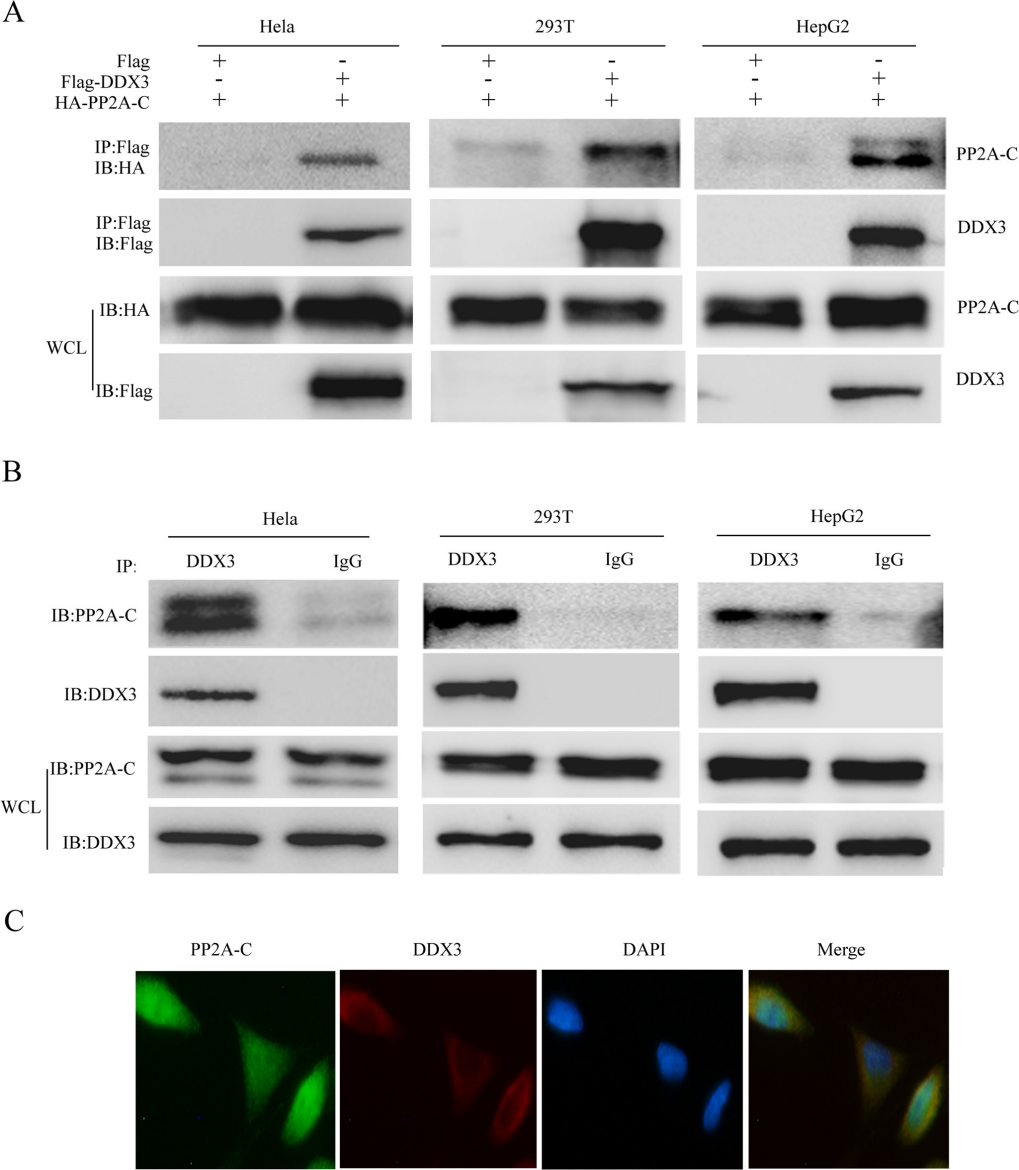
DDX3 interacts and colocalizes with PP2A-C HeLa, HEK-293T and HepG2 cells were first co-transfected with HA-tagged PP2A-C and Flag-tagged DDX3 expression plasmids for 48 hours; the cells were lyzed and the supernatant was immunoprecipitated with anti-Flag antibody. The result of coimmunoprecipitation and whole-cell lysates (WCL) were analyzed by immunoblot with an anti-HA or anti-Flag antibody (**A**). Immunoblot analysis of the interaction between endogenous DDX3 and PP2A-C in HeLa after immunoprecipitation with anti-DDX3 or mouse IgG. The whole-cell lysates (WCL) were also analyzed by immunoblot with an anti-DDX3 or anti-PP2A-C antibody (**B**). DDX3 colocalizes with PP2A-C. HeLa cells were first incubated with DDX3 antibody at 37°C for 1h before stained with Alexa Fluor^®^ 594 goat anti-mouse IgG (H+L) for 30min; the PP2A-C was incubated with PP2A-C antibody and stained with Alexa Fluor^®^ 488 goat anti-rabbit IgG (H+L) secondary antibody. The nucleus was stained with DAPI at 37°C for 5 min. Confocal images were obtained by a Zeiss LSM 710 scanning confocal microscope Data are representative of at least two independent experiments (**C**).

In order to study the mechanism on how DDX3 regulates the NF-κB signal pathway, we used the Flag-DDX3 as the bait to perform the immunoprecipitation experiment. The immunoprecipitated proteins were further analyzed by mass spectrometry (data not shown). In the list, we found the candidate protein, the catalytic subunit of the protein phosphatase 2A (PP2A-C). As a major serine/threonine phosphatase in eukaryotic cells [53], PP2A regulates many cellular events such as proliferation, survival and apoptosis [54]. Increasing evidence demonstrates that PP2A also regulates NF-κB signaling pathway [64, 65, 75]. Therefore, we suspected that PP2A-C subunit might regulate NF-κB signaling pathway through its interaction with DDX3. We next studied the possible interaction between DDX3 and PP2A-C subunit by co-immunoprecipitation in DDX3 and PP2A-C over-expressing cells, which confirmed that the Flag-tagged DDX3 interacted with HA-tagged PP2A-C subunit (Figure 4A). We further examined whether DDX3 and PP2A-C subunit interacted in physiological condition by immunoprecipitation of the endogenous proteins. We observed that PP2A-C subunit could be immunoprecipitated with the antibody specific to DDX3, but not with a control antibody (mouse immunoglobulin G) (Figure 4B). The interaction between DDX3 and PP2A-C was also confirmed in HEK-293T and HepG2 cells (Figure 4A, Figure 4B).

At last, we determined the subcellular localization of DDX3 and PP2A-C, which showed that DDX3 colocalized with PP2A-C in the cytoplasm under the normal physiological condition in HeLa cells (Figure 4C). Our result showed that DDX3 was mainly distributed in the cytoplasm which is consistent with other reports [24, 25, 32].

### PP2A-C regulate the NF-κB signal pathway by affecting the phosphorylation of p65 and IKK-β

**Figure 5:**
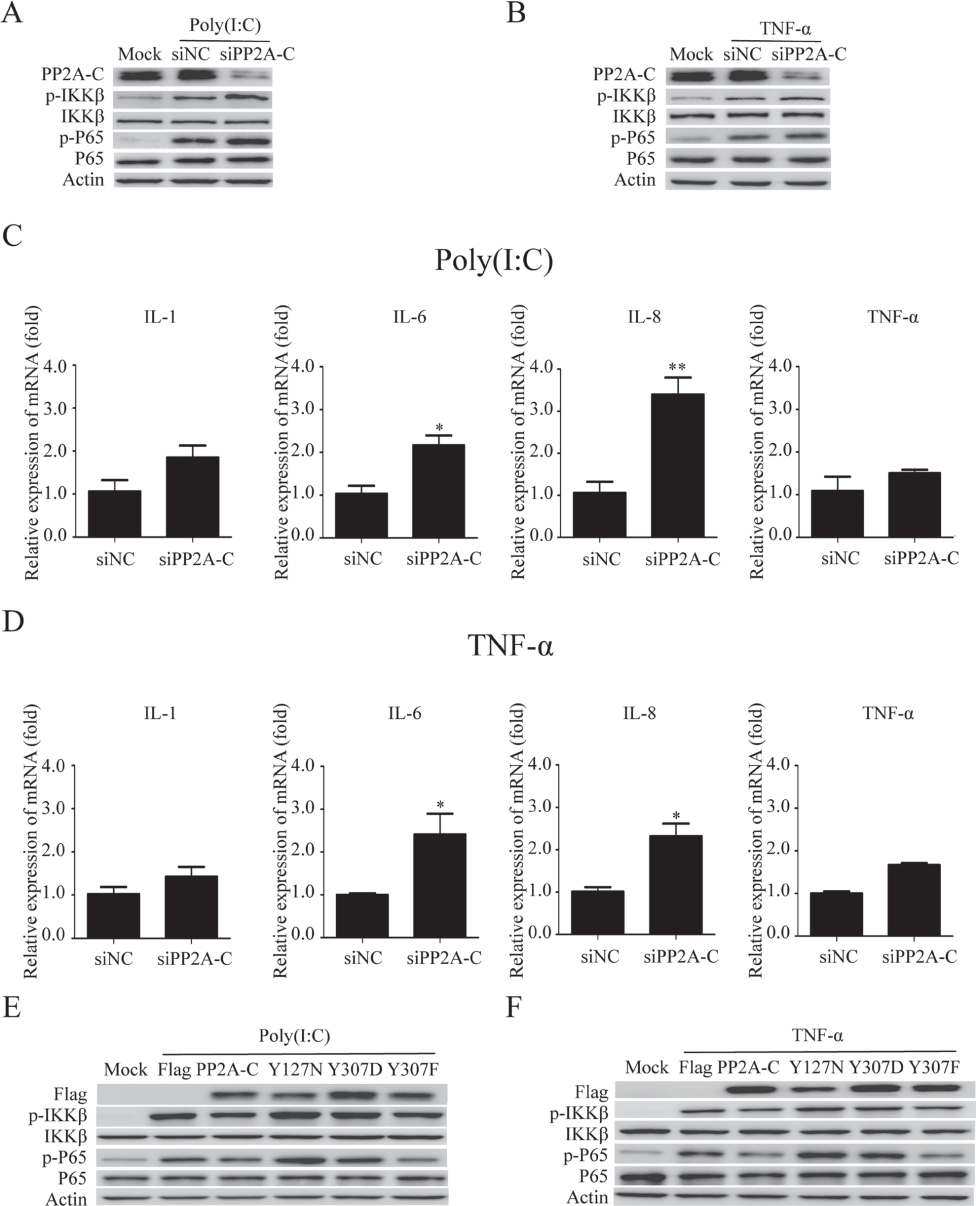
PP2A-C regulates the NF-κB signal pathway The PP2A-C expression was silenced with siPP2A-C in HeLa cells. The cells were transfected with poly(I:C)(5 μg/ml) or stimulated with TNF-α(25 ng/ml) for 6 hours. The phosphorylation of IKK-β and p65 were detected by western blot (**A, B**) and the relative mRNA levels of IL-1, IL-6, IL-8, TNF-α were assessed by qRT-PCR (**C, D**). The scrambled siRNAs (siNC) were used as the control. The differences between means were considered significant at *p < 0.05, very significant **p < 0.01. HeLa cells were transfected with the wild type PP2A-C and its mutants (Y127N, Y307D, or Y307F) for 48 hours, followed by poly(I:C)(5 μg/ml) transfection or TNF-α (25 ng/ml) stimulation for 6 hours. The phosphorylation of IKK-β and p65 were detected by western blot (**E, F**).

PP2A regulates NF-κB signaling pathway [65, 75]. Knockdown of PP2A-C expression with specific siRNA markedly attenuated PP2A activity [76]. Suppression of PP2A-C expression enhances the IKK-β phosphorylation induced by TNF-α, IL-1β, and LPS, as well as IL-6 gene expression, in HeLa cells [77]. We observed the similar results in our experiment, demonstrating that PP2A-C knockdown up-regulated the phosphorylation of p65 and IKK-β after poly(I:C) or TNF-a stimulation (Figure 5A and 5B). The mRNA of IL-1β, IL-6, IL-8, and TNF-α were also increased in PP2A-C knockdown cells (Figure 5C, 5D). The mRNA levels of IL-6 and IL-8 were increased significantly by 2.1-fold and 3.3-fold after stimulated with poly(I:C), whereas TNF-α stimulation increased mRNA levels of these two cytokines by 2.3-fold.

Over-expression of PP2A-C β-isoform results in de-phosphorylation of MEKK3 at Thr-516 and Ser-520 and termination of MEKK3-mediated NF-κB activation [77]. PP2A-C α-isoform over-expression attenuates JNK, ERK, PKC, and IKK phosphorylation, and impairs LPS/MCM-stimulated cell invasion and MCM-promoted cell growth [78]. Our results revealed that over-expressing PP2A-C reduced the phosphorylation of IKK-β and p65 in cells stimulated with poly(I:C) or TNF-α (Figure 5E, 5F). However, the over-expression of PP2A-C mutant (Y127N)[79], which was impaired in its catalytic activity, increased the phosphorylation of IKK-β and p65 (Figure 5E, 5F).

Methylation and phosphorylation are two major modifications that have been shown to modulate PP2A catalytic activity [80, 81]. Phosphorylation of Tyr^307^ on PP2A-C inactivates PP2A [82]. The phosphorylation-mimicking mutation PP2A-C (Y307D) [83] increased the phosphorylation of IKK-β and p65, whereas the non-phosphorylatable and constitutively-active mutation PP2A-C (Y307F) [83–85], reduced the phosphorylation of IKK-β and p65 (Figure 5E, 5F). All these results indicated that the catalytic activity of PP2A-C subunit regulated the NF-κB signal pathway by affecting the phosphorylation status of IKK-β and p65.

### DDX3 regulates the interaction between IKK-β and PP2A-C

**Figure 6:**
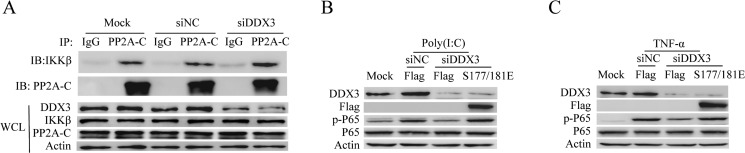
DDX3 regulates the interaction between IKK-β and PP2A-C HeLa cells were first transfected with siRNA (siNC or siDDX3) for 48 hours. The interaction between endogenous IKK-β and PP2A-C in HeLa cells was analyzed by the immunoprecipitation with anti-PP2A-C or rabbit IgG. The whole-cell lysates (WCL) were also analyzed by immunoblot with the anti-DDX3, anti-IKK-β, anti-PP2A-C or anti-actin antibody (**A**). DDX3 expression was first silenced before cells were transfected with the plasmids: Flag and Flag-IKK-β (S177/181E) for 48 hours. The cells were then transfected with poly(I:C)(5 μg/ml) (**B**) or stimulated TNF-α(25 ng/ml) (**C**) for 6 hours. The phosphorylation of p65 was detected by western blot.

Our above results revealed that DDX3 interacted with PP2A-C (Figure 4A, 4B). Several studies suggest PP2A-C interacts with IKK-β [64, 75, 86]. Therefore, we performed the co-IP experiment to confirm whether DDX3 formed a complex with PP2A-C and IKK-β. As expected, PP2A-C interacted with IKK-β (Figure 6A) [75, 86]. Meanwhile, we found that the interaction between PP2A-C and IKK-β was enhanced after DDX3 knockdown (Figure 6A). Phosphorylating IKK-β at Ser177 and Ser181 is a prerequisite for NF-κB activation [87, 88]. Numerous studies reveal that PP2A dephosphorylates IKK-β [62–64, 75], which was also verified in our studies (Figure 5A–5F). Since DDX3 knockdown affected the phosphorylation of IKK-β and attenuated the activation of NF-κB. We next determined whether the IKK-β mutant (S177/181E, the constitutively active mutant) could restore the decreased phosphorylation of p65 caused by the DDX3 knockdown. As expected, the over-expression of IKK-β mutant (S177/181E) indeed restored the decreasing tendency of phosphorylation of p65 (Figure 6B, 6C). All these data could explain why the silence of DDX3 expression reduced IKK-β phosphorylation and attenuated the subsequent NF-κB activation (Figure 2B–2D).

### DDX3 controls the phosphorylation of PP2A-C

**Figure 7:**
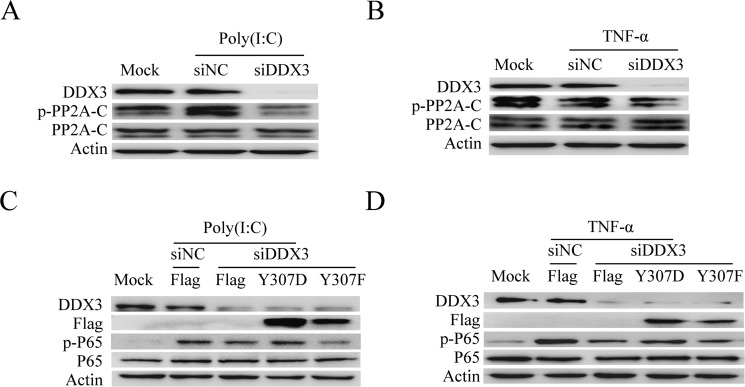
DDX3 controls the phosphorylation of PP2A-C DDX3 expression was silenced before the phosphorylation of PP2A-C was tested by western blot after the cells were either transfected with poly(I:C)(5 μg/ml) (**A**) or stimulated with TNF-α (25 ng/ml) (**B**) for 6 hours. DDX3 expression was first silenced before cells were transfected with the plasmids: Flag, Flag-PP2A-C(Y307D) and Flag-PP2A-C(Y307F) for 48 hours. The cells were then transfected with poly(I:C)(5 μg/ml) (**C**) or stimulated TNF-α(25 ng/ml) (**D**) for 6 hours. The phosphorylation of p65 was detected by western blot.

We have testified that the phosphorylation state of PP2A-C could affect the catalytic activity of PP2A and consequently influence the NF-κB signal pathway (Figure 5E, 5F). We next wondered whether DDX3 might regulate the NF-κB signal pathway by controlling the phosphorylation of PP2A-C. The phosphorylation of Tyr^307^ on PP2A-C inactivates PP2A [82]. Our western blot analysis showed that DDX3 knockdown reduced the phosphorylation of PP2A-C at Tyr^307^ (Figure 7A and 7B). We further determined whether the PP2A-C mutant (Y307D) was able to restore the decreased phosphorylation of p65 caused by the DDX3 knockdown. The non-functional mutant PP2A-C(Y307D) is the phosphorylation-mimicking mutation that abolished the phosphatase activity of PP2A [83]. Over-expression of this mutant increased the phosphorylation of IKK-β and p65 (Figure 5E, 5F). Based on our hypothesis, PP2A-C (Y307D) over-expression might bypass the effect of DDX3 knockdown. As expected, PP2A-C mutant (Y307D) indeed restored the decreased phosphorylation of p65 caused by DDX3 knockdown (Figure 7C, 7D). On the other hand, the non-phosphorylatable and constitutively active mutation PP2A-C (Y307F) [83–85], which reduced the phosphorylation of IKK-β and p65 (Figure 5E, 5F), could not restore the decreased phosphorylation of p65. In summary, these results indicated that DDX3 regulated NF-κB signal pathway by controlling the phosphorylation of PP2A-C.

## DISCUSSION

As a member of the DEAD-box helicase family, DDX3 plays important roles in RNA metabolism, apoptosis, and cancer. Meanwhile, DDX3 is one of the components of innate immune signaling pathways. Most studies about the DDX3 focus on its functions in IFN-β production. In this study, we reveal the involvement of DDX3 in NF-κB signal pathway regulation through protein phosphatase 2A.

The transcriptional levels of proinflammatory cytokines like IL-1β, IL-6, IL-8, and TNF-α were decreased after the DDX3 knockdown. It is well-known that the production of these cytokines was regulated by NF-κB. Our data confirmed that the degradation of IκBα, phosphorylation of p65, and the nuclear translocation of p65 were decreased after the DDX3 knockdown. Meanwhile, our experiment suggested that the ATPase and RNA helicase activities of DDX3 do not play significant roles in NF-κB signal pathway regulation.

The IKK complex consists of two catalytically active kinases (IKK-α and IKK-β) and a regulatory scaffold protein, NEMO [72, 73]. The IκB kinase complex (IKK) is necessary for IκB phosphorylation and degradation. In the canonical NF-κB pathway, IKK-β plays a greater role in activating complexes such as p50/RelA, p50/c-Rel [66]. Phosphorylating IKK-β at Ser177 and Ser181 results in IKK-β activation, followed by IκB is phosphorylation and degradation [87, 88]. Consistent with its effect on p65, our data demonstrated that DDX3 regulated the phosphorylation of IKK-β.

Our studies revealed that DDX3 interacted with protein phosphatase 2A C subunit. PP2A is a major serine/threonine PPase which can regulate many signaling pathways like Akt, p53, c-Myc and β-catenin [57–61]. The exact role that PP2A play in NF-κB signal pathway has not been fully confirmed, and there are even completely opposite views [86, 89]. Most of the reports show that PP2A negatively regulates NF-κB signal pathway [62, 63, 89], which was also supported by our studies.

PP2A has been shown to be a tumor suppressor by regulating many signaling pathways critical for cell transformation [90, 91]. The decreased activity of PP2A has been reported as a recurrent alteration in many types of cancer [92, 93]. Many endogenous inhibitors of PP2A, like CIP2A and SET, are emerging as key players in cancer cell survival and drug resistance [94, 95]. PP2A-C interacts with IKK-β and regulates IKK-β phosphorylation [64, 75]. We found that DDX3 exists in the complex of PP2A-C/IKK-β and the interaction between PP2A-C and IKK-β was enhanced after DDX3 knockdown, which might augment the dephosphorylation of IKK-β by PP2A-C.

PP2A-C can be subjected to two types of post-translational modification, phosphorylation and methylation. The phosphorylation of PP2A-C is catalyzed by p60v-src, p56lck, epidermal growth factor receptors, and insulin receptors; phosphorylation of PP2A-C occurred exclusively on Tyr^307^ *in vitro* [82]. PP2A also can rapidly reactivate itself in an auto-dephosphorylation reaction [96]. Phosphorylating PP2A-C on Tyr^307^ results in inactivation of the enzyme by inhibiting the interaction of PP2A-C with the PP2A-PR55/PR61 subunit [54, 83]. Our results showed that DDX3 regulated the phosphorylation of PP2A-C on Tyr^307^. On the other hand, PP2A-C mutant (Y307D) and IKK-β mutant (S177E/S181E) restored the decrease of p65 phosphorylation affected by DDX3 knockdown. These results strongly demonstrated that DDX3 might regulate NF-κB signal pathway by controlling the phosphorylation of PP2A C subunit. Another modification is methylation of PP2A by PP2A-methyltrans-ferase (PPMT). Leucine carboxyl methyltransferase 1 (LCMT1) methylates PP2A-C on Leu^309^ and regulate the ability of PP2A to form the heterotrimer with B subunit (B/PR55) [97, 98]. Whether DDX3 is involved in PP2A-C methylation and how DDX3 controls the phosphorylation of PP2A-C need further investigations.

NF-κB family members control the transcription of cytokines as well as genes that regulate cellular differentiation, survival and proliferation, inflammation and innate immunity. NF-κB is constitutively activated in many types of cancers due to the inflammatory microenvironment and various oncogenic mutations. In myeloid cells, activation of NF-κB increases the secretion of proinflammatory cytokines, such as TNF-α and IL-6, which enhance the inflammation and eventually leads to a rapid proliferation of tumor cells [99]. NF-κB signaling also contributes to cancer progression by controlling epithelial to mesenchymal transition, metastasis and the vascularization of tumors via upregulation of VEGF and its receptors [100, 101].

Several reports have shown that DDX3 is involved in tumor regulation. But the role of DDX3 in cancer development is controversial. Some researchers believe DDX3 has an oncogenic role in breast cancer and glioblastoma multiforme since it can regulate the cell adhesion and mobility by controlling E-cadherin promoter, and modulate transcription of Snail and Rac1-β-catenin signal to promote cell transformation and migration [27, 102, 103]. DDX3 also affects cell cycle progression by regulating Wnt/β-catenin-TCF4 signal to drive colorectal cancer [104]. Meanwhile, DDX3 can inhibit apoptosis that promote cancer cell proliferation [105, 106]. In medulloblastoma, DDX3 is one of the most frequently mutated genes [107]. Mutant DDX3 enhances cell viability and proliferation by combining with mutant β-catenin [107]. Recent studies have shown that the level of DDX3 in cells can be regulated by some proteins like Ezrin. Ezrin is a member of ERM (ezrin-radixin-moe-sin) family and interacts with DDX3. Ezrin maintains the intracellular protein level of DDX3 without changes in DDX3 mRNA [108]. Some researchers also claim that DDX3 acts as a tumor suppressive gene in colorectal cancer and hepatocellular carcinoma [23, 109]. DDX3 up-regulates the promoter activity of p21 ^waf1/cip1^ by promoting Sp1 to bind to p21 ^waf1/cip1^ promoter and leads to cell cycle arrest and apoptosis [26].

Although we revealed that DDX3 knockdown educed the phosphorylation of p65 and IKK-β and ultimately reduced the production of inflammatory cytokines induced by poly(I:C) or TNF-α stimulation the over-expression of wild-type DDX3 did not significantly affect the phosphorylation of p65 and the production of proinflammatory cytokines (Figure 3). One possible reason was that the endogenous DDX3 might be enough to regulate the interaction between PP2A-C and IKK-β (Figure 6A). Meanwhile, we transfected the wild-type DDX3, the biological function of mutant DDX3 shown in various tumor cells might not be observed in our studies.

In recent studies, DDX3 is considered as a novel target for antitumor agent development [110]. RK-33, a small molecule inhibitor designed to bind to DDX3 and inhibit its activity, can promote tumor regression [104, 111]. Ketorolac salt directly interacts with DDX3, which could be used to treat oral cancer [112]. The study of DDX3 regulating NF-κB signal pathway can provide theoretical guidance for the novel anti-cancer strategies.

A recently published article shows that DDX3 interacts with NF-κB subunit p65 and suppresses p65-mediated transcription [113], which was contradictory to our data presented here. In their studies, the authors claim that DDX3 interacts with p65 and suppresses p65-mediated transcription in 293T cells using luciferase reporter system; knockdown of DDX3 increases the mRNA levels of IL-6 and IL-8 in HepG2 cells after TNF-a stimulation. Therefore, we silenced the expression of DDX3 in HEK-293T and HepG2 cells and stimulated the cells with poly(I:C) and TNF-α. The western blot analysis showed the decreased phosphorylation of p65, which was consistent with our result in HeLa cells. Meanwhile, over-expression of DDX3 has no effect on the phosphorylation of p65 in HEK-293T and HepG2 cells.

In summary, our studies find that DDX3 exists in the complex of PP2A-C/IKK-β and regulates the interaction between IKK-β and PP2A-C. DDX3 controls the phosphorylation of PP2A-C, thereby affecting the phosphorylation of IKK-β. Our findings provide a new insight to explore the function of DDX3 and PP2A in innate immune system. The more precise roles of DDX3 in NF-κB signal pathway need to be further studied.

## MATERIALS AND METHODS

### Cell culture and stimulation

HeLa, HEK-293T, and HepG2 cells were maintained in Dulbecco's minimal essential medium (DMEM) (Gibco, USA) with 10% fetal bovine serum (FBS, Gibco, USA), penicillin (100 U/ml) and streptomycin (100 μg/ml) at 37°C in 5% CO_2_. HeLa or other cells were stimulated with poly(I:C) (Invivogen) and TNF-α (Life Technology). Poly(I:C) (1 μg/ml or 5 μg/ml) was transfected into the cells using TransIT-2020 Transfection Reagent (Mirus Bio) according to the manufacturer's instructions. TNF-α (5 ng/ml or 25 ng/ml) was diluted in the PBS and added directly to the medium.

### Plasmids, RNA interference and transfection

To construct Flag-tagged DDX3 and PP2A-C plasmids, DDX3 or PP2A-C gene was amplified from cDNA that was synthesized by reverse transcription using total cellular RNA isolated from HeLa cells. The DDX3, PP2A-C and IKK-β mutant plasmids, Flag-DDX3(K230E and S382L), Flag-PP2A-C(Y127N, Y307D and Y307F), and Flag-IKK-β (S177/180E) were prepared by fusion PCR.

RNA interference was used to knock down DDX3 and PP2A-C. DDX3 and PP2A-C small interfering RNAs (siDDX3 and siPP2A-C) were purchased from Biotend (Shanghai, China). HeLa or other cells grown to 60 to 70% confluence in plates were transfected with Lipofectamine 2000 transfection reagent (Invitrogen) according to the instruction of Lipofectamine 2000 transfection reagent. Briefly, plasmids or siRNA was diluted in serum-free Opti-MEM medium (Invitrogen). After incubation for 5 min at room temperature, the diluted plasmids or siRNA and Lipofectamine 2000 were mixed and incubated for 20min at room temperature. The mixtures were then added dropwise to each well. The plates were incubated at 37°C for 6 h, and the supernatants were removed. Cells were washed three times with PBS and incubated for an additional 48 h before the samples were used for other treatments. The primer sequences for PCR and the interference sequences for DDX3 and PP2A-C are listed in Table 1.

**Table 1:** The sequences for primers and siRNA

Name	Sequence
DDX3	Forward-GGCATGAAGCTTGCCACCATGAGTCATGTGGCAGTGGReverse-CATGCCGGATCCGTTACCCCACCAGTCAACCC
DDX3(K230E)	Forward-TGTGCCCAAACAGGGTCTGGAGAGACTGCAGCATTTCTGTTGCCCReverse-GGGCAACAGAAATGCTGCAGTCTCTCCAGACCCTGTTTGGGCACA
DDX3(S382L)	Forward-CGCCACACTATGATGTTTAGTGCTACTT TTCCTAAGGAAReverse-TTCCTTAGGAAAAGTAGCCAAAAACATCATAGTGTGGCG
PP2A-C	Forward-GATCCAAGCTTGCCACCATG GACGAGAAGGTGTTCACCAAGGAGCTGGReverse-GGATCGGATCCGGATCGGATCCCAGGAAGTAGTCTGGGGTACGACGAGTAACATGTG
PP2A-C(Y127N)	Forward-GAGAGCAGACAGATCACACAAGTTAATGGTTTCTATGATGAATGTTTAAGReverse-GGATCGGATCCGGATCGGATCCCAGGAAGTAGTCTGGGGTACGACGAGTAACATGTG
PP2A-C(Y307D)	Reverse-GGATCGGATCCCAGGAAATCGTCTGGGGTACGACGAGTAACATGTG
PP2A-C(Y307F)	Reverse-GGATCGGATCCCAGGAAAAAGTCTGGGGTACGACGAGTAACATGTG
IKKβ	Forward-GATCCAAGCTTGCCACCATGAGCTGGTCACCTTCCCTGACAACGCAGReverse-GGATCTCTAGATGA GGCCTGCTCCAGGCAGCTGTGC
IKKβ(S177/181E)	Forward-GATCAGGGCGAGCTTTGCACAGAGT TCGTGGGGACCReverse-GGTCCCCACGAACTCTGTGCAAAGCTCGCCCTGATC
siDDX3	Forward-CUGGCAACCUCAUUCUUUAdTdTReverse-UAAAGAAUGAGGUUGCCAGdTdT
siPP2A-C	Forward-CGCCAUCUAUAGAUACACUdTdTReverse-AGUGUAUCUAUAGAUGGCGdTdT

### Antibodies and other reagents

DDX3 rabbit polyclonal antibody (#11115-I-AP) was purchased from Proteintech (China). DDX3 mouse polyclonal antibody (sc-365768) and phospho-PP2A-C mouse monoclonal antibody (sc-271903) were purchased from Santa Cruz Biotechnology (USA). Antibodies for the proteins IKK-β(#2684), p-IKKα/β(S177/181)(#2697), NF-κB p65 (#8242), p-NF-κB p65 (Ser536)(#3033), IκBα(#4814), p-IκBα(#5209), PP2A-C Subunit (#2038), Histone H3(#4499) were purchased from Cell Signaling Technology(U.S.A). β-actin mouse monoclonal antibody (A1978), anti-Flag M2 mouse monoclonal antibody (F1804) and anti-HA rabbit antibody(SAB1306169) were purchased from Sigma-Aldrich. HRP-conjugated goat anti-rabbit or -mouse secondary antibodies were purchased from Jackson ImmunoResearch.

### RNA extraction and real-time quantitative RT-PCR (qRT-PCR)

The total RNA was extracted from cells with TRIzol reagent (Invitrogen) and purified according to the manufacturer's recommendation. For cDNA preparation, 2 μg of the total RNA was converted to cDNA using M-MLV Reverse transcriptase (Promega) with oligo(dT) primers in a total volume of 50 μl according to the manufacturer's recommendation. SYBR Green Master Mix (Vazyme, China) was used for qRT-PCR amplified following the manufacturer's protocol. The cellular β-actin mRNA from the same RNA extract was used as internal control. The copy number of the proinflammatory cytokines (IL-1β, IL-6, IL-8, TNF-α) was expressed as a ratio to cellular β-actin cDNA copies measured by qRT-PCR. qRT-PCR reactions were performed using the CFX96 Real-time PCR system (Bio-Rad).

### Western blot analysis

After removing the medium, cells were washed thoroughly with ice-cold PBS. Then the cells were harvested in lysis buffer (50 mM Tris-HCl, pH 7.4, 150 mM sodium chloride, 1% NP40, 1 mM EDTA, 5 mM sodium fluoride, 0.25% sodium deoxycholate, 5 mM sodium orthovanadate, 0.4% SDS, 1 mM PMSF) containing protease inhibitor and phosphatase inhibitor (Thermo, USA) for 2 h at 4°C. The concentration of proteins was determined by the Bradford assay then cell lysates were subjected to 10% or 12% SDS-PAGE and transferred to nitrocellulose membranes (Whatman International, Ltd.). The membrane was blocked with 5% non-fat milk powder in TBST buffer (20 mM Tris–HCl, pH 7.4, 150 mM NaCl, 0.1% Tween 20) for 1h at room temperature then was incubated with primary antibodies at 4°C overnight. The membrane was incubated with HRP-conjugated secondary antibodies for 1 h at room temperature, the antibody-antigen complex on the membrane was visualized by enhanced chemiluminescence system(Thermo).

### Co-immunoprecipitation assay

The cells were washed thoroughly at least 3 times in cold PBS and lysed for 2 h at 4°C in lysis buffer (50 mM Tris–HCl, pH 7.4, 150 mM NaCl, 1 mM EDTA, 0.2 mM PMSF, 1% NP-40) containing protease inhibitor and phosphatase inhibitor. The cell debris was removed by centrifugation at 10,000 × g for 10 min at 4°C, and the supernatant was collected. The supernatant was pre-cleared using the protein G agarose beads (Invitrogen) for 1 h at 4°C. The supernatant was then mixed with DDX3 mouse polyclonal antibody and incubated at 4°C overnight. The protein G agarose beads were then added to the mixture and incubated at 4°C for 75 min. The beads were washed five times with 1 ml washing buffer (20 mM Tris–HCl, pH7.5, 150 mM NaCl, 0.1% NP-40), and resuspended in 1×loading buffer and boiled. The protein samples were then subjected to Western blot analysis.

### Immunofluorescence staining and confocal microscopy

HeLa cells were seeded on cover slips in a 6-well plate for 24 h. The cells were transfected with siDDX3 to knockdown the expression of DDX3 for 48 h before stimulated with Poly(I:C) or TNF-α. The cells were washed three times with PBS, and fixed with 4% paraformaldehyde for 15 min. After blocked with 5% bovine serum albumin for 1 h at 37°C, the slips were incubated with p65 antibody at 37°C for 1 h before incubated with Alexa Fluor^®^ 488 goat anti-rabbit IgG (H+L) (Life Technology)for 30min. The nucleus was stained with DAPI (Beyotime Biotechnology) at 37°C for 5 min. Confocal images were obtained by a Zeiss LSM 710 scanning confocal microscope. To determine the colocalization of DDX3 and PP2A, HeLa cells were first incubated with DDX3 antibody at 37°C for 1h before incubated with Alexa Fluor^®^ 594 goat anti-mouse IgG (H+L) for 30 min. After blocked with 5% bovine serum albumin, the PP2A-C protein was stained with PP2A-C antibody and Alexa Fluor^®^ 488 goat anti-rabbit IgG (H+L). The nucleus was stained with DAPI at 37°C for 5 min. Confocal images were obtained by a Zeiss LSM 710 scanning confocal microscope.

### Statistical analysis

All experiments presented were repeated at least three separate times. The data represented the means ± standard deviations of triplicate determinations. All data was analyzed by one-way ANOVA using the SPSS 17.0 software package (version 17.0, SPSS Inc., Chicago, IL, USA). The differences between means were considered significant *p < 0.05, very significant at **p < 0.01.
